# Progress in immune microenvironment, immunotherapy and prognostic biomarkers in pediatric osteosarcoma

**DOI:** 10.3389/fimmu.2025.1548527

**Published:** 2025-01-22

**Authors:** Lin Zhang, Haoming Jiang, Haichao Ma

**Affiliations:** ^1^ Department of Orthopedics, The Third Affiliated Hospital of Shenzhen University, Shenzhen, China; ^2^ Department of Pediatrics, Shenzhen University General Hospital, Shenzhen, China

**Keywords:** pediatric osteosarcoma, immunosuppressive environment, immunotherapy, biomarkers, ICB, PD-1

## Abstract

Pediatric osteosarcoma, the most prevalent primary malignant bone tumor in children, is marked by aggressive progression and a generally poor prognosis. Despite advances in treatment, including multi-agent chemotherapy, survival rates remain suboptimal, with metastasis, particularly to the lungs, contributing significantly to mortality. The tumor microenvironment plays a crucial role in osteosarcoma progression, with immune cells such as tumor-associated macrophages and T lymphocytes significantly influencing tumor behavior. The immunosuppressive environment, dominated by M2 macrophages, contributes to immune evasion and poor therapeutic outcomes, though recent findings suggest the potential for reprogramming these cells to enhance immune responses. This review provides a comprehensive overview of the immune landscape in pediatric osteosarcoma, with a focus on the role of immune cells and their interactions within the tumor microenvironment (TME). It examines the impact of immune checkpoints, genetic mutations, and inflammatory pathways on osteosarcoma progression, highlighting their contribution to tumor immune evasion and disease advancement. Additionally, emerging immunotherapeutic strategies, such as immune checkpoint inhibitors, macrophage reprogramming, and antibody-based therapies, are summarized in detail, showcasing their potential to improve therapeutic outcomes.

## 1 Introduction

Pediatric osteosarcoma is the most common primary malignant bone tumor in children, representing a major challenge in pediatric oncology (1). It typically originates in the metaphyseal regions of long bones, notably the distal femur (43%), proximal tibia (23%), and humerus (10%) (2). The disease is highly aggressive, progressing rapidly with poor prognosis and high mortality rates despite treatment advancements. Pulmonary metastasis is the predominant site of distant spread, occurring in over 85% of metastatic cases, followed by bone metastases (3). Recurrence and metastasis are the main factors contributing to mortality and poor outcomes in pediatric osteosarcoma (3). Over the past four decades, multi-agent chemotherapy has yielded only modest survival improvements (4–7). Although risk factors for recurrence, such as tumor location and histological response to neoadjuvant chemotherapy, have been identified (2, 8, 9), risk-adapted therapies have largely failed to enhance outcomes (5, 10, 11). Consequently, there is an urgent need to identify new, modifiable prognostic factors to guide treatment strategies and develop novel therapies (12, 13).

Inflammation is a key feature in cancer biology, with the tumor microenvironment (TME) central to tumor progression and metastasis (14, 15). Immune cell interactions within the TME are pivotal, as evidenced in melanoma and breast cancer, where immune profiles correlate with patient outcomes and therapy responses (16, 17). The osteosarcoma TME comprises immune cells, osteoblasts, endothelial cells, stromal cells, extracellular matrix, and signaling molecules (14). Predominantly, tumor-associated macrophages (TAMs) with an M2 phenotype are present, alongside T lymphocytes, myeloid cells, and dendritic cells (18, 19). While M2 TAMs generally associate with poor 5-year event-free survival, some studies suggest they may reduce metastasis and improve survival in specific contexts (20). Thus, targeting immune components in the osteosarcoma TME emerges as a promising therapeutic strategy (21).

This review summarizes the current understanding of the immune microenvironment in pediatric osteosarcoma, exploring the potential immunotherapies and prognostic biomarkers that may guide future treatment approaches. By elucidating the role of immune cells, and their influence on disease progression, this review provides new insights into the pathogenesis of osteosarcoma and highlight opportunities for developing more effective and individualized therapies.

## 2 Immune microenvironment in pediatric osteosarcoma

### 2.1 The role of the immune system in osteosarcoma development

The immune system plays a crucial role throughout all stages of diseases (22, 23), with immune dysregulation significantly contributing to cancer initiation and progression (24). In OS, the immune system interacts with the TME in a complex manner. Tumor-infiltrating T cells, which are integral to anti-tumor immune responses, are activated within the TME (25, 26). However, these T cells often upregulate inhibitory receptors on their surface. When these receptors bind to corresponding ligands expressed by tumor cells, immune activity is suppressed. This immune suppression leads to a diminished anti-tumor immune response, allowing tumor cells to escape immune surveillance (27). Immune checkpoint blockade (ICB) therapy, which aims to disrupt these receptor-ligand interactions, has shown promise in enhancing T cell function and improving the immune system’s ability to target and eliminate cancer cells (28). Numerous clinical studies have demonstrated the efficacy of ICB in treating a variety of cancers (29–31), further stimulating research into the immune landscape of osteosarcoma. These findings underscore the importance of the TME in osteosarcoma, as it is closely associated with clinical outcomes, prognosis, and the response to immunotherapy (32, 33). Understanding the immune system’s role within the TME of osteosarcoma is critical for advancing therapeutic strategies aimed at improving outcomes for patients with this aggressive bone tumor (34).

### 2.2 Major infiltrating immune cell types in pediatric osteosarcoma

The immune microenvironment in osteosarcoma is complex, characterized by diverse immune cell infiltration, though complete characterization remains elusive (35–37). Macrophages and T cells are the predominant immune cells within osteosarcoma (38–41). Elevated levels of infiltrating macrophages and CD8^+^ T cells correlate with reduced metastasis and improved survival in osteosarcoma patients (42–44). Conversely, increased infiltration of antigen-presenting cells, such as dendritic cells, correlates with poorer clinical outcomes (45). Natural killer (NK) cells play a critical role in innate immunity by directly killing tumor cells through stress-induced ligands and absence of MHC class I molecules on tumor surfaces (46). However, the osteosarcoma microenvironment often impairs NK cell function through various immunosuppressive mechanisms, including the secretion of inhibitory cytokines and the expression of immune checkpoint molecules (47). Enhancing NK cell activity through cytokine therapy or adoptive cell transfer is being explored as a potential therapeutic strategy in pediatric osteosarcoma (46).

Besides, osteosarcoma cells release PD-L1 to suppress immune responses (48) and indoleamine 2,3-dioxygenase (IDO) inhibiting neoantigen generation by dendritic cells (DCs), facilitating immune escape (49). DCs are essential for antigen presentation and T cell initiation but are compromised by the tumor-induced immunosuppressive environment, such as TGF-β and IL-10, which inhibit DC maturation and antigen-presenting capacity, reducing effective T cell activation (50). Strategies to restore DC function, including DC vaccines and agents blocking immunosuppressive signals, are under investigation to enhance anti-tumor immunity in osteosarcoma (50). Additionally, all-trans retinoic acid (ATRA) suppresses macrophage M2 polarization, inhibiting lung metastasis (51). Signaling pathways like VEGF, IL-10A, TGF-β, and STAT3 modulate the immunosuppressive microenvironment by affecting suppressive cells, macrophages, and stromal fibroblasts (52). These findings underscore the pivotal role of the immune microenvironment in osteosarcoma prognosis and therapeutic outcomes.

Further analysis highlights both homogeneity and heterogeneity within the osteosarcoma immune microenvironment (53). This may partly explain the limited efficacy of current immunotherapies in osteosarcoma, as the TME is dominated by immunosuppressive M2 or non-functional M0 macrophages, hindering effective immune responses. Patients with CD8^+^ T cell infiltration may derive greater survival benefits from immunotherapies. Thus, inducing the transition of M0 or M2 macrophages to the pro-inflammatory M1 phenotype represents a promising strategy to enhance anti-tumor immune responses in pediatric osteosarcoma (54). Understanding immune cell dynamics within the osteosarcoma TME offers new avenues for more effective immunotherapeutic approaches.

### 2.3 Immune-related mechanisms in pediatric osteosarcoma

Understanding the immune-related mechanisms is crucial for developing targeted immunotherapies for pediatric osteosarcoma. Recent studies have identified differential expression of immune-related genes (IR-DEGs) within the TME, predominantly upregulated and enriched in various immune pathways (27). Macrophages play a pivotal role in the TME by interacting with other immune cells, promoting tumor development and progression while also contributing to tumor suppression through phagocytosis (55). Furthermore, T-cell cytotoxicity is vital for eliminating tumor cells, with tumor-infiltrating macrophages modulating T-cell activity, thereby impacting cancer prognosis and immunotherapy efficacy (56–58). Genetic mutations in key tumor suppressor genes, such as TP53, ATRX, and RB1 are extensively associated with osteosarcoma (59–61). Wu et al. found that mutations in these genes are more frequent in low-immunity groups, correlating with increased metastasis (27). Sexual dimorphism in immune responses has been observed, with significant differences in pathways related to macrophages, T-cells, B-cells, Th1, Th2, and the complement system (62, 63). Additionally, sex-specific differences are noted in the PD-1/PD-L1 immunotherapy pathway and signaling pathways such as calcium, p53, and cell cycle regulation, suggesting gender-tailored therapies may enhance outcomes (62).

**Figure 1:**
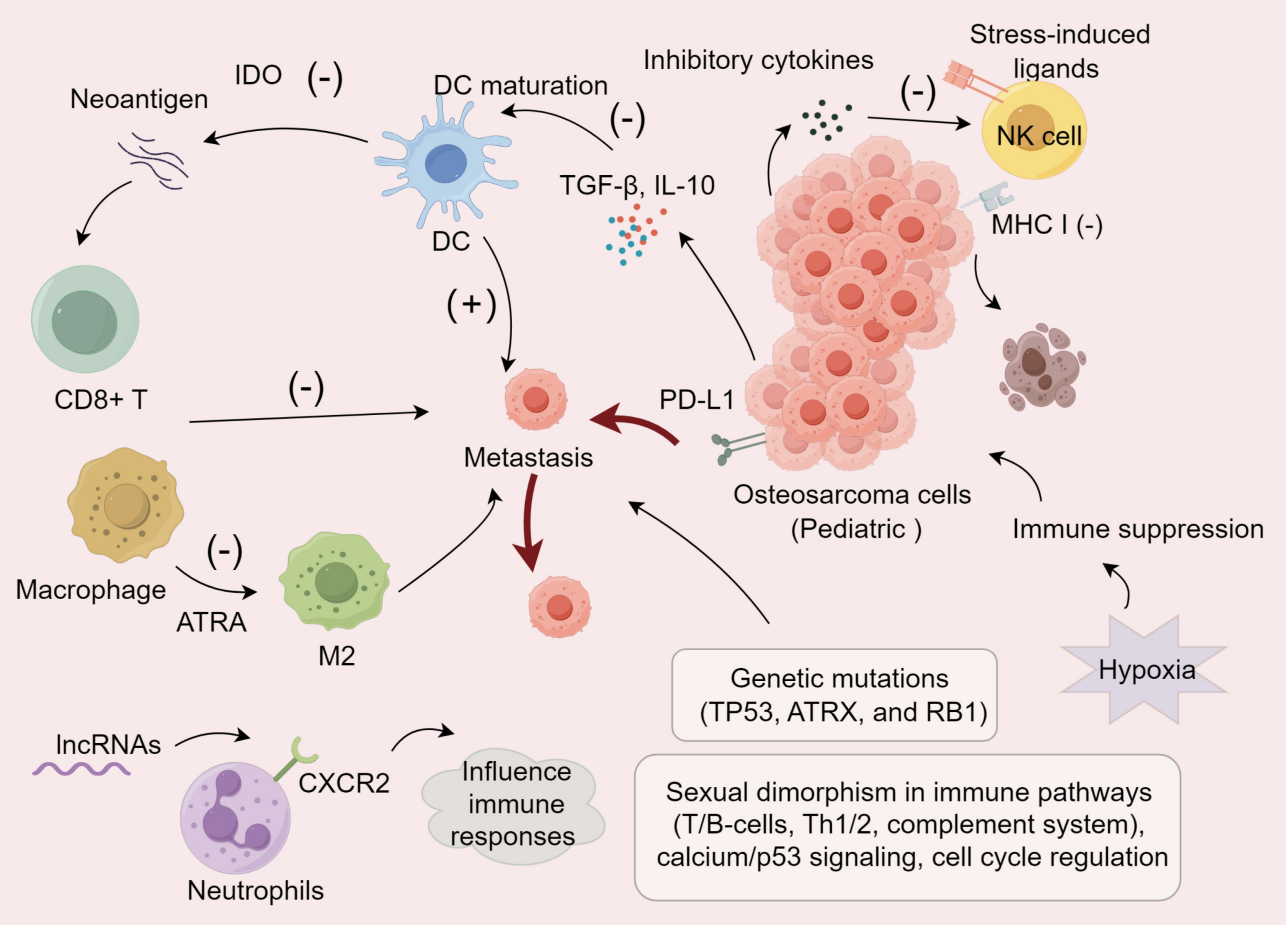
Immune modulation mechanisms within the pediatric osteosarcoma TME.

Long non-coding RNAs (lncRNAs) are crucial regulators in osteosarcoma, affecting immune cell infiltration in the TME by modulating immune-related gene expression. A novel modeling algorithm (64) indicated that metastatic osteosarcoma patients have reduced activation of memory CD4^+^ T cells, monocytes, mast cells, and neutrophils compared to localized cases. irlncRNAs) may influence immune responses by regulating chemokine receptors like CXCR2 in neutrophils (65), and facilitating non-classical monocyte migration to the lungs, a primary metastasis site (66, 67). Hypoxia, common in malignant tumors, significantly impacts osteosarcoma progression by inducing immune cell death and impairing immune responses within the TME (68), including T-cell and NK cell activation, thereby fostering an immunosuppressive environment (69, 70). Moreover, hypoxia enhances VEGF expression, promoting tumor advancement (71). In conclusion, the immune landscape of pediatric osteosarcoma is complex, involving macrophages, T-cells, genetic mutations, gender differences, hypoxia, and lncRNAs, collectively contributing to tumor progression and immune evasion, thus offering novel strategies for improving osteosarcoma prognosis (**Figure 1**).

## 3 Advances in immunotherapy for pediatric osteosarcoma

Osteosarcoma treatment traditionally encompasses chemotherapy, surgery, and radiation, with methotrexate-based chemotherapy forming the cornerstone of systemic therapy, supplemented by agents such as doxorubicin, and cisplatin. Immunotherapy, an innovative approach in cancer treatment, aims to bolster the immune system’s capability to combat cancer progression and establishment (72).

### 3.1 L-MTP-PE

The most compelling evidence for immune modulation in osteosarcoma comes from the use of mifamurtide [liposomal muramyl tripeptide phosphatidylethanolamine (L-MTP-PE)] (72). Approved for use with standard treatment regimens in non-metastatic osteosarcoma, it raised the 6-year survival rate from 70% with chemotherapy alone to 78% (73–75). L-MTP-PE activates macrophages and monocytes, leveraging the immune system’s cancer-fighting capabilities (74). Punzo et al. observed a slowdown in OS progression through the activation of macrophages by L-MTP-PE and notably, its direct action on MG63 cells. They demonstrated not only the anti-tumor activity of L-MTP-PE in OS but also induced a shift in macrophage phenotype from M1/M2, promoting a balance between pro-inflammatory and immunoregulatory functions (76). L-MTP-PE also influences bone metabolism, inducing anti-osteoporotic effects in children undergoing chemotherapy for osteosarcoma (77).

### 3.2 Antibody therapies in pediatric osteosarcoma

Antibody therapies like olaratumab, in combination with doxorubicin, has been approved as first-line for soft tissue sarcomas (78), and carotuximab with pazopanib in Phase III trials for angiosarcomas (79), are gaining ground in osteosarcoma treatment. Glembatumumab-vedotin targets osteoactive substances overexpressed on osteosarcoma cells, showing cytotoxic effects in both osteosarcoma and breast cancer (80–83). Trastuzumab, initially for HER2^+^ breast cancer, targets HER2 tyrosine kinase activity critical for cell proliferation, which is present also in osteosarcoma cells, indicating its potential for osteosarcoma therapy (84, 85). Research with nivolumab in humanized mouse models has revealed that while the primary tumor volume and growth rate of osteosarcoma matched the control group, the rate of lung metastases was significantly reduced, underscoring the potential of targeting the tumor microenvironment with immunotherapy (54).

### 3.3 Innovative immunotherapy strategy

Recent advances in immunotherapy have shown promise in the treatment of pediatric osteosarcoma, leveraging both innovative antibody technologies and the enhancement of immune system responses. A novel approach involves the use of bispecific antibodies, specifically anti-CD3 x anti-GD2, which recruit T cells to significantly enhance the tumor-killing effects of anti-GD2 antibodies (86). Additionally, the combination of chemotherapy and immunotherapy, such as irinotecan and temozolomide with denosumab, has demonstrated significant activity in neuroblastoma, suggesting its potential applicability in osteosarcoma (87, 88).

### 3.4 Chimeric antigen receptor T-cell therapy

CAR-T therapy represents a cutting-edge immunotherapeutic approach that has revolutionized the treatment of certain hematological malignancies and is now being explored in solid tumors, including pediatric osteosarcoma. CAR-T therapy involves the genetic modification of a patient’s T cells to express receptors that specifically target tumor-associated antigens (89). Potential targets for CAR-T therapy include HER2, GD2, and B7-H3, which are overexpressed on osteosarcoma cells (90). Preclinical studies have demonstrated the efficacy of CAR-T cells in recognizing and eliminating osteosarcoma cells *in vitro* and *in vivo* (91). Clinical trials are currently underway to evaluate the safety and efficacy of CAR-T cell therapy in pediatric osteosarcoma patients (92). Ongoing research is focused on enhancing the persistence and infiltration of CAR-T cells within the TME in osteosarcoma, as well as overcoming the immunosuppressive barriers that limit their efficacy (92).

### 3.5 Combinations of immunotherapy and chemotherapy

Recent strategies include liposomal and aerosolized drug formulations such as sustained-release lipid inhalation targeting cisplatin and aerosolized granulocyte–monocyte colony-stimulating factor (GM-CSF) alongside NK cell infusions and aerosol IL-2, enhancing local chemotherapy efficacy for lung metastases in metastatic osteosarcoma (93). IL-2 activates lymphocytes into lymphokine-activated killer (LAK) cells, effective against multidrug-resistant cells and targeting lung metastasis sites (94, 95), underlining the potential of IL-2 and LAK/NK cell-based therapies in managing pediatric osteosarcoma lung metastases. In addition, denosumab, targeting the receptor activation of nuclear factor kappa-β ligand (RANKL), reduces fracture risks in tumor metastases and showed a 99% inhibition in giant bone cell tumor progression in Phase II studies (96, 97). The RANK-L/RANK/osteoprotegerin (OPG) pathway plays a critical role in osteosarcoma, with studies indicating its involvement in tumor progression and potential as a therapeutic target (98–101). Despite these advances, challenges remain, particularly in managing the drug’s impact on standard chemotherapy effectiveness and deciphering the mechanisms behind resistance to immunotherapy (102–104). Thus, there is a pressing need to identify effective combinations of immunotherapy and conventional treatments to overcome resistance pathways and enhance therapeutic outcomes in pediatric osteosarcoma.

## 4 Prognostic factors in pediatric osteosarcoma

### 4.1 Clinical and traditional prognostic indicators in pediatric osteosarcoma

Accurate prediction of prognosis is related to determining the best treatment plan for an individual (105, 106). Therefore, accurate prognostic indicators are crucial for optimal treatment strategies in pediatric osteosarcoma. Traditional markers like Enneking surgical standards and alkaline phosphatase often show variability across the same tumor stages, however lacking precision (107–110). Histological subtype and tumor stage are considered inaccurate and inadequate prognostic parameters (3). Critical adverse prognostic factors include age, gender, tumor size, metastases at diagnosis, and poor chemotherapy response, highlighting the insufficiency of traditional prognostic methods (111–115). Notably, metastasis at diagnosis is a major independent prognostic factor (116, 117). Other reported adverse factors include high serum alkaline phosphatase levels, involvement of the visceral pleura. Additionally, the histological response to neoadjuvant chemotherapy is recognized as a prognostic factor for both metastatic and non-metastatic patients (93). Delay in time to completion of chemotherapy is independently associated with poor prognosis in children with osteosarcoma (118).

### 4.2 Immune, inflammatory, metabolic, and genetic prognostic factors

The immunological characteristics of the osteosarcoma TME are valuable for prognosis. TAMs and T cells play a key role in determining cancer prognosis and the efficacy of immunotherapies (27). Inflammation markers such as the Systemic Immune-Inflammation Index (SII) are potent predictors of tumor prognosis, offering new directions for predicting survival at different time points and improving long-term survival rates (3). A higher Immune-Score correlates with better survival due to greater immune infiltration, whereas higher Tumor-Purity correlates with lower survival rates (27). Metabolic factors also significantly impact prognosis. Ma et al. have shown that preoperative platelet-to-albumin ratio (PAR) and apolipoprotein B-to-apolipoprotein ratio (ApoB/ApoA1) are independent prognostic factors for 5-year overall survival (105). On the genetic front, Yang et al. identified 69 DEGs related to metastasis and immune infiltration, with GATA3, LPAR5, EVI2B, RIAM and CFH demonstrating prognostic potential (119). Mutations in TP53 and high levels of WNT6, resulting from low DNA methylation, are associated with poor pediatric osteosarcoma prognosis (120, 121). Additionally, circulating tumor DNA (ctDNA) detected by next-generation sequencing (NGS) offers novel prognostic insights for localized bone tumors (122). The multivariate Cox model identified miR-29b and miR-422 as independent prognostic markers, where their downregulation predicts poor outcomes, underscoring their value in pediatric osteosarcoma prognosis (123).

In addition, the presence of a four-gene signature associated with hypoxia (EFNA1, P4HA1, STC2, and MAFF) correlates with clinical and molecular features and is an important prognostic predictor in pediatric osteosarcoma patients (71). Human osteosarcoma cells have exosomes that express specific metalloproteinases (MMP-1 and -13) that are involved in cell recruitment and cancer cell colonization and are therefore good therapeutic targets or good biomarkers for prognosis (72). There is a strong correlation between circulating angiogenic factors and poor prognosis (124). In a Cox proportional hazards model, VEGF-A expression in biopsy samples was confirmed to be an independent prognostic factor for poor survival in osteosarcoma (125) (**Table 1**). Integrating clinical, immune, inflammatory, metabolic, and genetic prognostic factors enables personalized treatments and better risk stratification for pediatric osteosarcoma. Challenges include standardizing biomarker assessments, advanced bioinformatics, interdisciplinary collaboration, large-scale validation, ethical issues, and cost-effectiveness. Overcoming these barriers is essential for enhancing patient outcomes through targeted therapies and immunotherapies.

**Table 1:** Prognostic marker in pediatric osteosarcoma.

Category	Specific Factor(s)	Association with Prognosis
Clinical and Traditional Indicators	Enneking surgical standards, Alkaline phosphatase	Variable across tumor stages, lacking precision
	Histological subtype, Tumor stage	Inaccurate and inadequate prognostic parameters
	Age, Gender, Tumor size, Metastases at diagnosis, High serum alkaline phosphatase levels, Involvement of the visceral pleura, Chondroblastic subtype	Adverse prognostic factors; metastasis at diagnosis is a major independent adverse prognostic factor; others also associated with poor outcomes
	Histological response to neoadjuvant chemotherapy	Prognostic for both metastatic and non-metastatic patients
	Delay in time to completion of chemotherapy (TCC)	Independently associated with poor prognosis
Immune, Inflammatory, Metabolic, and Genetic Biomarkers	Tumor-associated macrophages (TAMs), T cells, Immune-Score, Tumor-Purity	Key roles in prognosis and efficacy of immunotherapies; Higher Immune-Score correlates with better survival; Higher Tumor-Purity correlates with lower survival rates
	Systemic Immune-Inflammation Index (SII)	Potent predictor of tumor prognosis
	PAR, ApoB/ApoA1, ApoA1 levels	PAR and ApoB/ApoA1 are independent prognostic factors for 5-year overall survival; Lower ApoA1 levels associated with worse survival through macrophage transformation and enhanced tumor-promoting inflammation
	Differentially expressed genes (GATA3, LPAR5, EVI2B, RIAM, CFH)	Show prognostic potential
	Mutations in TP53, ATRX, RB1; WNT6 levels due to low DNA methylation	Associated with OS development, invasion, metastasis; Higher mutation frequency in low-immunity group linked to higher metastasis; High WNT6 levels associated with poor prognosis
	Circulating tumor DNA (ctDNA); miR-29b and miR-422	ctDNA provides prognostic information in localized bone tumors; miR-29b and miR-422 are independent prognostic markers for overall survival, with downregulation linked to poor prognosis
	Four-gene signature associated with hypoxia (EFNA1, P4HA1, STC2, MAFF); Exosomes expressing MMP-1 and MMP-13; Circulating angiogenic factors; VEGF-A expression in biopsy samples	Hypoxia gene signature is an important prognostic predictor; MMP-expressing exosomes are therapeutic targets or biomarkers; Circulating angiogenic factors correlate with poor prognosis; VEGF-A is an independent prognostic factor for poor survival

## 5 Conclusions

In conclusion, pediatric osteosarcoma remains a challenging malignancy with poor prognosis despite advances in treatment. The TME, particularly the immune landscape, plays a crucial role in disease progression and metastasis. TAMs, T lymphocytes, and other immune cells interact within the TME, influencing both immune evasion and therapeutic response. Emerging immunotherapies, including immune checkpoint inhibitors and novel antibody treatments, show promise in enhancing anti-tumor immunity. However, overcoming immunosuppressive factors, such as M2 macrophage polarization and immune checkpoint upregulation, is essential for improving outcomes. Identifying prognostic biomarkers within the TME could guide individualized treatment strategies and ultimately improve survival rates in pediatric osteosarcoma patients. Furthermore, future research should prioritize optimizing specific immunotherapeutic strategies, such as enhancing the efficacy of immune checkpoint inhibitors and macrophage-targeted therapies; developing personalized treatment plans tailored to individual patient’s immune profiles and genetic backgrounds; and designing robust clinical trials to evaluate the safety and effectiveness of these novel approaches.
